# Is the Virus Important? And Some Other Questions

**DOI:** 10.3390/v10080442

**Published:** 2018-08-19

**Authors:** Ruth-Anne Sandaa, Gunnar Bratbak

**Affiliations:** Department of Biological Sciences, University of Bergen, Bergen N-5006, Norway; Gunnar.Bratbak@uib.no

**Keywords:** Van Etten, chlorella virus, *Phycodnaviridae*, *Paramecium*, copepods

## Abstract

The motivation for focusing on a specific virus is often its importance in terms of impact on human interests. The chlorella viruses are a notable exception and 40 years of research has made them the undisputed model system for large icosahedral dsDNA viruses infecting eukaryotes. Their status has changed from inconspicuous and rather odd with no ecological relevance to being the *Phycodnaviridae* type strain possibly affecting humans and human cognitive functioning in ways that remain to be understood. The Van Etten legacy is the backbone for research on *Phycodnaviridae*. After highlighting some of the peculiarities of chlorella viruses, we point to some issues and questions related to the viruses we choose for our research, our prejudices, what we are still missing, and what we should be looking for.

## 1. Introduction

To meet the (often narrow-minded) call for relevance and benefit requested by funding agencies and politicians, most of us justify our research by claiming that the viruses we work on are important and that the work provides knowledge that will benefit society. The importance of a virus is not due to the virus itself, but to the hosts they infect and affect, and many viruses are important because they cause diseases in humans, animals, or crops. They are also important because they are active and abundant in aquatic environments, infect key species, and affect community composition and nutrient flow and thus all aquatic ecosystem services. Hence, we need to know about viruses to understand nature and implement knowledge-based management of our resources.

## 2. The Chlorella Viruses

Most of us work on “important viruses”, but some scientists have made a career working on viruses that when first isolated must have seemed peculiar and unimportant by any reasonable argument. The chlorella viruses (for reviews, see [1,2,3,4]) can only be propagated in previously symbiotic zoochlorellae isolated from, e.g., the protozoan *Paramecium bursaria* or the heliozoon *Acanthocystis turfacea*, and not in any naturally free-living *Chlorella* strains. The zoochlorellae are not found free-living in natural waters, where they are vulnerable to viral infection, but only inside their hosts, where they are protected. Different chlorella viruses are nevertheless found worldwide in aquatic habitats, so they must have a host in natural systems that is different from symbiotic zoochlorellae, or a way to circumvent the protection provided the host by being intracellular. Since about 1980, it has indeed been a paradox that the algal-type virus we know best has had an unknown natural host. The enigma does, however, now seem to have found a solution [5,6]. Presumably driven by chemotaxis, protists containing zoochlorellae appear to be attracted by chlorella viruses, which thus accumulate on their surface. Then when the protists are consumed by zooplankton, the cells are ruptured, while the zoochlorellae remain intact and unprotected against virus infection when defecated. Hence, zooplankton grazing makes infection possible and stimulates virus production. From an ecological point of view, this is truly a neat system of interactions. It broadens our conception of viral activity in natural systems, suggesting that viruses together with their microbial hosts may also affect other organisms (e.g., by chemical signaling) and modify their behavior in ways that directly or indirectly ensure virus proliferation.

Although the chlorella viruses and their hosts may not be important per se, they are easy to grow and maintain in culture and thus are excellent model systems for large icosahedral dsDNA viruses that replicate in eukaryotes. These peculiar systems have revealed a wealth of information on the molecular properties and morphology of these large DNA viruses (reviewed by [1,2,3,4]). There have certainly been many surprising findings including the number of genes, the presence of DNA site-specific endonucleases that cleave only one strand of dsDNA, and the small size of many enzymes (e.g., ATP-dependent DNA ligase [7], type II DNA topoisomerase [8], K^+^ channel [9], histone metyltransferase [10]. Moreover, some chlorella viruses encode for biosynthesis of chitin and hyaluronan, which both accumulate on the external surface of their infected cells [11,12]. Chitin is otherwise associated with the cell wall of fungi and the exoskeletons of crustaceans and insects but in light of the recent finding that zooplankton (and maybe also insect?) grazing plays a role in virus proliferation, the production of chitin and chitinases might make sense. Production of hyaluronan, which in addition to the capsules of a few pathogenic bacteria is only found in vertebrates, where it is associated with the extracellular matrix in, e.g., connective, epithelial, and neural tissues, is not as easily rationalized. The staggering discovery that algal virus genes are apparently present in some human brains and may affect human cognitive functioning [13] might indicate that there is a connection between chlorella viruses and vertebrates where a hyaluronan-covered surface is important, but the most common chlorella virus sequences found in humans (virus ATCV-1) does not have a hyaluronan synthesis gene [14]. The chlorella host–virus system is weird, discovered serendipitously but insightfully recognized as a great model system that has taught us a lot about viruses, that may have ecological relevance, and that may affect humans in ways that remain to be understood. If not important by itself when discovered, it has indeed been made important by the Van Etten legacy.

## 3. Open Issues and Unanswered Questions

The extensive basic work on chlorella host–virus systems forms a solid and very important basis for the more ecology-oriented generation of virologists that prospered around the turn of the century. Delbrück and The Phage Group selected and confined their attention to the seven T-phages [15]. This was a very successful strategy, but with the obvious drawback that the properties the T-phages lack, such as lysogeny, were disregarded for many years. Van Etten and co-workers focused on technically simple and (in the laboratory) well-behaved host–virus systems and a few viruses. The question is now what we, basing much of our knowledge on algal host–virus systems that are generally easy to isolate and well-behaved, are overlooking?

The hallmarks of virus switching to a new host species are acute infections with high host mortality [16,17]. Further evolution of any ecologically successful parasite will ensure host survival rather than mortality and viruses may hence be expected to evolve from causing acute infections with high mortality towards less virulent and more persistent infections [17,18]. Many viruses and their respective hosts have presumably co-existed and evolved in the ocean for eons, so persistent infections [19,20] should therefore be the rule, whereas acute infections with high mortality are the exception. Does this mean that many of the viruses we have isolated (e.g., Emiliania huxleyi virus (EhV), Phaeocystis pouchetii virus (PpV) and P. globosa virus (PgV)) that are causing mass mortality in the ocean and eye-catching symptoms in cultures are the odd ones and that the typical average ones remain to be isolated? Our virus isolation procedures select neither for slow-growing forms with long latency time, low burst, and incomplete lysis, nor for viruses with a size or shape (e.g., filamentous) that is excluded in any prefiltration step. What about non-lethal infections where the virus is secreted from the living cell: will they be found with the protocols we use? There is indeed still a lot to discover and learn. Chance favors the prepared mind!

Another issue, prompted by the chlorella virus natural host question discussed above, relates to host range and naming of algal viruses. Common practice is to name the virus “Genus species Virus” according to the species used as host for isolation. In most cases, host range is tested using a few related strains and species, not anticipating the possibility that the species used may be just an accidental victim of viral infection, while the main host in situ belongs to another genus or family, or may be something completely different, e.g., Crustacea, Ciliophora, or even Vertebrata. Viruses able to proliferate in more than one genus, such as some Haptolina ericina (HeV RF02) and Prymnesium kappa (PkV RF01 and RF02) viruses [20], may perhaps be common in aquatic ecosystems as the ecological advantage seems obvious. The regular and ecologically important host(s) in situ should ideally be identified before a new virus is named. To make things more complicated, the taxonomy of phytoplankton, on which we depend to name new viruses, is also being revised; e.g., *Chrysochromulina ericina* is now *Haptolina ericina* [21]. The virus (CeV01B [22]) has, however, not changed its name. Moreover, the nomenclature for naming organisms implies relatedness, but this does not apply to viruses. Two viruses infecting the same host may be phylogenetically widely different (e.g., PkV RF01 and PkV RF02 [20]), while viruses infecting different hosts may be quite similar and phylogenetically related (e.g., PgV, CeV01B, PpV, and Pyrimimonas orientalis virus (PoV)). A revision of the nomenclature we use for naming our algal viruses is thus warranted.

In 1989, Van Etten wrote, “The ecological role that these viruses play in nature is completely unknown. Unfortunately, the water ecologists I have talked to have no interest in the viruses”. (Personal letter to GB after reading the *Nature* paper on high abundance of viruses found in aquatic environments). He may still be partly right about the ecological role of chlorella viruses, but when it comes to algal viruses in general and the water ecologists, he is now completely wrong. Thanks, Jim!
